# Eye anomalies in children born through ART

**DOI:** 10.22336/rjo.2021.65

**Published:** 2021

**Authors:** Andreea Mădălina Bănică, Simona Daniela Popescu, Simona Vlădăreanu

**Affiliations:** * “Carol Davila” University of Medicine and Pharmacy, Bucharest, Romania; Department of Neonatology, “Elias” University Emergency Hospital Bucharest, Romania

**Keywords:** eye anomalies, assisted reproductive techniques, ophthalmological screening

## Abstract

Due to the increasing rate of couples suffering from infertility, recently, the use of assisted reproductive technology (ART) has increased by 5%–10% per year. Some ART pregnancies are at risk of obstetric and neonatal complications, but it is unknown whether the procedures used or the etiology of infertility influences this risk. Multiple ART pregnancies are responsible for the increased rate of prematurity and its associated complications. Also, it has been demonstrated that ART pregnancies have a higher risk of congenital anomalies. The rate of ART-associated ocular abnormalities is incompletely known due to a small number of studies conducted regarding this pathology. In this paper, we presented a review of literature on ocular anomalies associated with ART in order to raise awareness of the need to implement ophthalmological screening in children from pregnancies obtained by ART.

**Abbreviations:** ART = assisted reproductive techniques, IVF = in vitro fertilization, ICSI = intracytoplasmic sperm injection, LBW = low birth weight, ROP = retinopathy of prematurity

## Introduction

Although the use of assisted reproductive technology (ART) to treat infertility is becoming more common nowadays [**1**], the follow-up programs of these children are incompletely developed worldwide. This is because not enough studies that follow the long-term prognosis of these patients have been conducted [**2**]. Data from literature show that there is an association between ART and the risk of birth defects, especially in the case of multiple pregnancies [**3**], but there are still controversies [**4**]. Recently, awareness of ART pregnancies has increased, and studies have shown an association with congenital heart abnormalities [**5**-**8**], urogenital anomalies [**3**,**9**,**10**], anomalies of the central nervous system [**11**], but the visual system was poorly studied.

## Risk of congenital anomalies in ART pregnancies

The mechanisms by which congenital anomalies occur in these patients are not completely elucidated but it is considered that certain procedures used in assisted reproduction contribute to this risk. Some studies show that intracytoplasmic sperm insemination (ICSI) increases the risk of congenital anomalies more than in vitro fertilization (IVF) [**12**,**13**]. The association between ICSI and the risk of malformations has been shown to be closely linked to male subfertility [**14**,**15**]. Thus, a population cohort study demonstrated that the use of ART increases the risk of non-chromosomal abnormalities in singleton pregnancies and chromosomal abnormalities in multiple pregnancies, mainly obtained through ICSI [**12**]. However, in a retrospective study, no significant differences were demonstrated between these two techniques [**16**]. Another study showed that there was no significant difference between the two methods when it comes to birth defects, except for hypospadias, which was more frequently correlated with ICSI [**17**].

Another mechanism related to the occurrence of congenital anomalies is the alteration of DNA methylation process [**18**,**19**]. DNA methylation errors may occur during the embryonic preimplantation period, in the context of using micromanipulation techniques. Thus, in an epidemiological study, a 4.46-8.91 times higher probability of occurrence of Beckwith-Wiedemann syndrome and Silver Russell syndrome in the ART group was demonstrated [**19**]. 

Taking these data into account, studies have reported that patients diagnosed with congenital anomalies have a higher risk of cancer, including those obtained through ART. The hazard ratio is higher for IVF children with cancer, especially if they were diagnosed with chromosomal defects [**20**]. However, these results are not generally valid as a follow-up study of children from IVF pregnancies compared to those from pregnancies of subfertile women did not show significant differences for the risk for cancer [**21**]. 

In most studies, the risk of birth defects was compared between pregnancies obtained through ART and those obtained spontaneously. However, studies have shown that there are additional factors that may influence the prognosis of these children. Thus, the cause of infertility, other parental characteristics, including maternal age, but also the comparison with the subfertile population of women who were diagnosed with infertility, but who did not need treatment to obtain a pregnancy, were considered [**22**]. All these factors influence both complications in pregnancy and the risk of birth defects in newborns obtained through ART. 

## Eye abnormalities associated with the use of ART

Congenital anomalies reported in the ART population involve damage to the central nervous system, cardiovascular, genitourinary system, but also the auditory and visual system [**13**]. 

Worldwide, the number of cases diagnosed with congenital anomalies in the ART population is not known, neither is the prevalence of eye abnormalities. 

ART was associated with eye malformations, reduced visual acuity and cases of anisometropia [**23**]. These abnormalities may be secondary to *de novo* deletions, which occurred early during the embryogenesis process, but may also be related to risk factors that influence the development of these pregnancies. In this regard, the manipulation of oocytes and sperm, the medication used for ovarian stimulation, the culture medium, and the temperature to which developing embryos are exposed may contribute as risk factors [**24**].

The development of the visual system begins early in pregnancy, between 3 and 10 weeks of gestation, and, at that moment, estrogen and progesterone receptors are expressed at the eye level. Ovarian stimulation therapy from IVF protocols can cause changes at this level, with an exaggerated increase in the concentration of these hormones [**25**]. Thus, the fetus is vulnerable to develop eye abnormalities. This theory was tested in a prospective study, which showed that children obtained through IVF with controlled ovarian stimulation protocols were more likely to require eyeglasses at 11 years old compared to the control group (23% versus 11%) [**26**]. The need for ovarian stimulation to achieve a pregnancy demonstrates that certain parental characteristics that contributed to infertility may influence the prognosis of these pregnancies. Regarding this matter, a study showed that children obtained by IVF have a similar risk of visual disturbances compared to the general population after adjusting for possible confounding factors (maternal age, parity, smoking status, nutritional status) [**27**]. On the contrary, a recent systematic review did not demonstrate significant differences in the risk for abnormalities in morphology and ocular function in the ART group due to the low number of high-quality studies. For this reason, evidence from large, prospective studies is needed to assess the risk and generalize a conclusion [**28**]. 

The use of ART increased the incidence of multiple pregnancies, due to the frequent transfer of at least 2 embryos, which correlates with the increased incidence of prematurity in this population group [**29**,**30**]. 

The increase in long-term morbidity in children from IVF pregnancies was mostly correlated with the high rate of prematurity in these pregnancies, but this only partially explains the associated complications [**31**]. Studies have shown that preterm infants are at risk of retinopathy of prematurity (ROP), reduced visual acuity, refractive errors, strabismus, and amblyopia [**24**,**32**]. Two recent retrospective studies have attempted to demonstrate the association between the use of ART and the risk of ROP. The results show that, although newborns from ART pregnancies do not have a higher risk of ROP compared to those obtained spontaneously [**33**,**34**], they have more severe forms of the disease [**33**]. The associated prematurity and the low number of studies makes it difficult to establish exactly the cause that leads to ocular complications in ART children. 

Literature data report an increased frequency of refractive errors in ART children, including myopia and hyperopia [**24**,**35**], with no difference for astigmatism [**23**]. As procedural risk factors, the use of ICSI does not change the frequency of refractive errors in these children compared to the general population [**36**,**37**].

In a retrospective study, ART children had more frequently red reflex abnormalities, and 20.3% of them, especially premature babies, were diagnosed with poor fixation control. These results correlate with the increased incidence of refractive errors in the ART group mentioned above, which, with time, leads to impaired visual acuity [**24**].

The frequency of major eye abnormalities was also analyzed in this at-risk population. In a retrospective study, it was shown that 26% of newborns obtained through IVF had major eye abnormalities, compared with those born spontaneously. Of these, congenital cataracts, and glaucoma, hypoplastic optic nerves, retinoblastoma, and optic nerve coloboma have been reported. The anomalies were mostly unilateral, sporadic, without detection in the antenatal screenings [**35**]. 

Given that the studies reported a higher incidence of congenital anomalies after ICSI [**12**,**13**], a prospective study was conducted to assess the risk of auditory and visual abnormalities in this population. Children with a mean age of 5.5 years from the ICSI group were compared with those obtained spontaneously. No significant differences were identified between the two groups for the studied effects. 28% of children in the ICSI group were diagnosed with visual impairment, compared with 35% in the control group [**37**]. On the contrary, a study demonstrated the association between the use of ICSI and the risk of abnormal retinal vascularization in boys. The reduced number of retinal branching points remained significant even after adjusting for possible confounders (prematurity, low birth weight - LBW) [**38**]. These contradictory results highlight that the use of ICSI alone does not increase the risk of ocular abnormalities, but possibly its association with other risk factors, including parental characteristics, may influence the results.

Studies correlating the risk of malignancy of ART children with congenital anomalies [**39**,**40**] have been investigating the incidence of retinoblastoma cases in this population group [**41**-**44**]. Retinoblastoma is a malignant disease, most commonly caused by two mutations in the *RB1* gene [**45**]. Medication for ovarian stimulation may cause hypermethylation of the RB1 gene promoter and this can partially explain the association between ART and retinoblastoma [**45**]. However, this theory has not always been statistically significant [**46**]. Two studies have shown that for an estimated IVF birth rate of 1.0% -1.5%, the relative risk of retinoblastoma is high, but the authors consider the results “a chance finding”. This is because the incidence of retinoblastoma was increased only for a limited period (1996-2002) [**41**,**42**]. These results are supported by a study that did not show the association between IVF and the risk of retinoblastoma [**44**]. More studies are needed to demonstrate this association. 

## Conclusion

The development of the visual system begins early in utero, and it can be influenced by internal factors, correlated, in the context of ART, with micromanipulation technology, but also with the medication used in controlled ovarian stimulation. For this reason, children obtained through ART should be included early in vision-screening programs. Periodical ophthalmological risk assessment and counseling of children diagnosed with eye diseases, both antenatal and postnatal, should be performed in the first 12 months of life. Currently, due to a low percentage of studies on this topic, there are no concrete protocols and indications for screening and follow up of these patients. 

**Conflict of Interest statement**

None. 

**Acknowledgements**

None. 

**Sources of funding**

None. 

**Disclosures**

None.
